# Peer support during pregnancy and early parenthood: a qualitative study of models and perceptions

**DOI:** 10.1186/s12884-015-0685-y

**Published:** 2015-10-12

**Authors:** Jenny McLeish, Maggie Redshaw

**Affiliations:** Policy Research Unit for Maternal Health and Care, National Perinatal Epidemiology Unit, University of Oxford, Old Road Campus, Headington, Oxford, OX3 7LF UK

**Keywords:** Peer support, Pregnancy, Early parenthood, Perinatal, Volunteer, Lay, Non-professional, Models, Perceptions

## Abstract

**Background:**

Peer support is a flexible concept used in healthcare across diverse areas to describe the activities of individuals acting in a non-professional capacity offering support to others with whom they have some experience in common. There is little research on peer supporters and women supported in the context of the transition to parenthood and disadvantage. This study particularly focuses on peer support for women experiencing a range of vulnerabilities during pregnancy and the postnatal period, in projects which assigned trained volunteers to individual pregnant women. There were three core elements to the volunteers’ support in these projects: active listening, providing information, and signposting to local services in the area. Many also offered practical support.

**Methods:**

This was an descriptive qualitative study, informed by phenomenological social psychology, exploring experiences and perceptions of giving and receiving voluntary peer support during pregnancy and early parenthood in England, with a particular focus on disadvantaged women. Participants took part in semi-structured, audio-recorded interviews, the transcripts of which were analysed using thematic analysis.

**Results:**

Forty-seven volunteers and 42 mothers were interviewed, from nine peer support projects. The overarching themes identified were (1) ‘What is peer support?’, containing two themes: ‘befriending or mentoring’, and ‘responding to the individual’; (2) ‘Who is a peer supporter?’, containing two themes: ‘someone like me’, and ‘valuing difference’; (3) ‘The peer support relationship’, containing five themes: ‘a friend or a ‘professional friend’, ‘building relationships of trust’, ‘avoiding dependency’, ‘managing endings’, and ‘how peer supporters differ from professionals’.

**Conclusion:**

A variety of models of volunteer peer support have been offered to pregnant women and new mothers in England. All create a structure for meaningful relationships of trust to occur between volunteers and vulnerable women. In the absence of agreed definitions for the nature and boundaries of peer support during pregnancy and early parenthood, it is important that projects provide clear information to referrers and service users about what they offer, without losing the valued flexibility and individuality of their service.

## Background

Peer support is a flexible concept, used in relation to healthcare across a number of fields including mental health, chronic illness, breastfeeding and the transition to parenthood. It has been broadly used to describe the activities of people acting in a non-professional capacity to offer support to others with whom they have some experience in common. They may be paid workers [1] or volunteers [2, 3]; they may be trained as part of an organised scheme [2, 3] or simply be other service users [4]; they may support others by visiting them [2, 3], telephoning them [5], attending a group with them [6] or connecting with them online through an internet forum [7]. The core theoretical construct underlying peer support has been described by Mead and MacNeil: “In general, peer support has been defined by the fact that people who have like experiences can better relate and can consequently offer more authentic empathy and validation. It is also not uncommon for people with similar lived experiences to offer each other practical advice and suggestions for strategies that professionals may not offer or even know about.” [8]

A number of different types of organised volunteer peer support have been developed for pregnant women and women during the months after birth, usually as part of small scale, time-limited projects. These peer support activities may target a specific issue or be aimed at women who are seen as ‘vulnerable’ in a variety of ways. Mothers and babies in disadvantaged circumstances (including being poor, isolated, a single mother, a young mother, a recent migrant, a refugee or asylum seeker) and mothers and babies from Black, Asian and ethnic minority groups have been found to have poorer physical and mental health outcomes [9–12] than other mothers and babies. They are also less likely to access maternity and child health services [13, 14]. Peer support interventions in England aiming to reduce these inequalities have included breastfeeding peer supporters who try to motivate others to breastfeed and support them in doing so [15]; ‘doulas’ who visit a mother frequently during pregnancy and up to three months after birth and support her during labour [16]; community parents who visit monthly (usually postnatally) and support mothers in setting and achieving goals for themselves [17]; and volunteers who support women experiencing perinatal anxiety and depression [18].

Athough volunteer home visiting services for families with young children are well established in the UK and Ireland [2, 17, 19], there has been increasing interest from English policymakers and funders in the capacity of the voluntary sector, and peer support schemes in particular, to contribute to maternal and child well-being during pregnancy and very early parenthood. In 2009, the Department of Health funded the roll-out of a volunteer doula programme at up to eight replication sites [16], and in 2011 the Department of Health’s Health and Social Care Volunteering Fund funded the charity NCT to run a four site pilot of a peer support scheme that covered pregnancy and the first two years after birth [20], and also funded the charity Refugee Council to run a four site Health Befrienders Network for refugees and asylum seekers, one site of which focused on pregnant women [21]. In 2010 the National Institute for Health and Care Excellence (NICE) recognised the role that voluntary organisations could play in the support of pregnant women with complex social needs, and recommended research into the effectiveness of voluntary sector family support on improving outcomes for mothers and babies [10].

Most pregnancy and early parenthood peer support projects cited above aim to create behaviour change and improve outcomes for mothers and children. However, there is a very limited research literature directly on women’s perceptions of peer support during pregnancy and afterwards. Small et al. note [22] in their discursive paper describing several studies involving professional and mother- to-mother social support interventions, that when recipients’ own opinions are reported, less emphasis is given to the information and educational aspects of the intervention. Instead they value non-judgemental companionship, reassurance and feeling less alone, although this is somewhat contradicted by Granville & Sugarman [3], who found that mothers valued having someone to rely on, good information, and a greater sense of control. That raises the question of what ‘peer support’ really means to those who deliver it and those who receive it.

This paper describes original qualitative interview-based research that explores what organised peer support during pregnancy and early parenthood means to volunteers who are trained to offer it and to women who receive it. It first describes some of the current models in practice in England and then explores volunteers’ and mothers’ views about what the peer supporter does, what it means to be a peer, how the relationship works, and where the boundaries of the role lie for both groups.

## The context

The research was carried out at nine peer support projects in England targeting disadvantaged women during pregnancy and the postnatal period. The projects were diverse: their characteristics are shown in Table 1. Some offered support to vulnerable pregnant women and new mothers in a defined geographical area or under the care of a specific hospital; some worked with target groups defined by their circumstances such as young mothers, mothers from Black, Asian and minority ethnic communities, mothers who were asylum seekers and refugees, or mothers with very complex needs; others worked with pregnant women who had a specific health issue such as women living with HIV or women experiencing mental health problems. Most were in urban settings (in Bristol, Bradford, Burnley, Halifax, Huddersfield and London) and one served rural communities in and around an army base. The length of the peer support varied, with most projects accepting referrals at any stage of pregnancy and formally stopping support at time points ranging from when the baby was between six weeks to two years of age.

**Table 1:** Characteristics of the nine peer support projects

	Primary target group	Location	Type of peer support	Period of support	Support to access services	Practical/material support	Set-up	Host	Initial Training for volunteers	Current Funding
1	Women with very complex needs	London	Small team of volunteers	Pregnancy, at birth, to 12 weeks postnatal (longer if needed)	yes	yes	1996	Local charity	40 h (8 sessions)	Charitable
2	Women in local area	London	1:1	Pregnancy to 3 months postnatal (longer if needed)	yes	yes	2006	Local community development charity	36 h	Public
3	First time mothers in local area	London	1:1	Pregnancy to 8 months postnatal	yes		2012	Local community development charity	72 h (18 sessions) - City & Guilds level 3	Partly charitable, partly public
4	Women receiving maternity care from a specific hospital trust	London	1:1	Pregnancy to 12 months postnatal	yes	yes	2012	Local community development charity	8 h (4 sessions)	Charitable
5	Refugees and asylum seekers	North of England	1:1, 2:1	Pregnancy to 2 years postnatal	yes	yes	2011	National charity	30 h – Open College Network Level 2	Public; ended 2014
6	Young women and women experiencing difficult circumstances	North of England	1:1, groups	Pregnancy to 2 years postnatal	yes		2012	National charity. In 2014 became an independent Community Incorporated Organisation.	30 h – Open College Network Level 2	Public; then charitable.
7	Women from minority ethnic communities; young women	North of England	1:1, groups	Pregnancy to 2 years postnatal	yes	yes	2011	National charity	30 h – Open College Network Level 2	Public; ended 2014.
8	Women living with HIV	London	1:1, group	Pregnancy and short period postnatal; 6–12 visits	yes	yes	2010	National charity	36 h (6 sessions) –Open College Network Level 2	Charitable
9	Women with depression/anxiety	West of England	1:1, group	Group has 12 sessions; 1:1 for 6 weeks (more if needed)			2011	Local charity	In development	Charitable

There was considerable variation in how the projects delivered peer support. The most common method was 1:1 support with a trained, unpaid volunteer allocated to a mother as her peer supporter, but sometimes two or more volunteers would support a mother together. Some projects also ran groups to bring mothers together for mutual support and sometimes therapeutic activities, and one provided support at birth as well as during pregnancy and limited postnatal support. Some took a community development approach that included empowerment and social support for both supported mothers and the volunteers.

Eight projects had a formal training programme that volunteers had to complete before working with mothers (the ninth was developing its training). This initial training ranged between four and eighteen sessions and varied between 8 and 75 contact hours. In most projects it could lead to an accredited qualification for the volunteers. Project-co-ordinators also arranged ongoing training for their volunteers and gave them regular supervision.

In some projects the support provided by volunteers was expected to be intensive, with contact every week and sometimes more frequently immediately after birth (when there could be daily visits); in others it was much less intensive (visits once a month and occasional phone calls). To some extent volunteers were expected to agree a visiting pattern with the mothers they supported, so there could be wide variation within a project depending on the volunteers’ other commitments.

In all the projects there were three core elements to the volunteers’ support: firstly, active listening; secondly, providing information about pregnancy, birth and parenting, about the English healthcare system and other official systems; and thirdly, signposting to local services in the area. In most of the projects the volunteers also offered a fourth element: material and practical support. This could include providing mothers with second hand baby clothes and equipment, accompanying them to medical appointments and interpreting for them, acting as birth partners, helping to resolve benefits and housing issues, cooking for them and helping with childcare, giving them money for a taxi fare to hospital to give birth, liaising with immigration authorities and social services, and accompanying them to children’s centre groups, food banks, and other services and activities.

## Methods

### Study design

This was an experiential qualitative descriptive study [23], based on semi-structured, in-depth interviews, informed by the theoretical perspective of phenomenological social psychology [24], and underpinned by contextualism [25]. This “low-inference” [23] design was chosen because the purpose of the study was to explore participants’ own perceptions and lived experiences, and thus to stay close to their accounts without superimposing a theoretical framework or generating theory [24], while acknowledging the role of both participants’ understandings and the researchers’ interpretations in the production of knowledge [26] and the researchers’ responsibility to ground findings closely in participants’ actual experiences [27].

The Oxford University Medical Sciences Research Ethics Committee (reference MSD-IDREC-C1-2013-111) approved the study.

### Data collection

The projects were identified following contact with a voluntary groups association and a national charity which focuses on pregnancy, birth and the early years of parenthood. The projects were chosen to reflect a diversity of geographical locations and target populations. Projects that were currently or had recently been involved in independent research were excluded to avoid participant and organisation burden. Data from two projects were excluded from the analysis: one where professional counselling was offered and a second where the drop in nature of the support did not facilitate identification of supported women. A researcher (JM) contacted the co-ordinator of each peer support project by telephone to introduce the research and establish whether the project was able to participate. One project co-ordinator declined on behalf of his organisation because of the need to prioritise fundraising. The researcher arranged face to face interviews with each co-ordinator in order to gain an understanding of the individual project and to describe the research aims and process. These interviews provided contextual information about the project’s objectives and functioning and were not analysed as part of this research study. The co-ordinator then described the research to the project’s volunteers and supported mothers using the study information leaflets (one version for mothers and one for volunteers) and asked their permission for the researcher to contact them, or arranged with those who wished to participate when the researcher could interview them. The sampling was thus purposive insofar as all participants had experience of giving or receiving peer support. One volunteer and one supported woman who had agreed to their contact details being passed on, decided not to participate when contacted by the researcher, citing logistical problems (an unstable housing situation and a young baby).

Between July 2013 and September 2014, the researcher travelled to meet each person who agreed to participate at the project base, at their home, or at another place of their choice. Having obtained written informed consent, a semi-structured qualitative interview based on the topic guide designed for volunteers or mothers as appropriate was carried out. The interviews explored the participant’s experience of giving or receiving peer support and her experience of maternity services. Topics for mothers included: their involvement with the peer support project, the impact of peer support, and their feelings about the voluntary nature of the peer support and its ending. After the first six interviews, the topic guide was modified to pursue an emerging theme on specific peer experiences. Topics for volunteers included the training, peer support activities, support received from the project, the impact on supported women and the impact on the volunteer. The duration of interviews varied (mothers: range 17–90 min, median 44 min; volunteers: range 20–89 min, median 49 min) Two interviews were particularly short as a consequence of unrelated practical difficulties which included childcare.

All but two interviews were carried out face to face, with one mother and one volunteer being interviewed by telephone where it was not possible to arrange such a meeting (oral informed consent was given and recorded). On two occasions, two volunteers were interviewed together at their request. Professional interpreting for participants whose first language was not English was offered, however, none of the participants took up the offer. One interview was informally interpreted by the mother’s peer supporter at the mother’s request. All the participant interviews were audio-recorded and fully transcribed.

### Data analysis

The mothers’ and volunteers’ interviews were analysed as separate data sets. The interview transcripts were analysed using inductive thematic analysis [28]. Each verbatim transcript was first checked against the audio recording, and then by reading and rereading each transcript, codes were identified inductively and recorded using NVIVO software. Codes were refined, combined and disaggregated as data collection continued, and emergent themes identified; the technique of constant comparison [29] was used to reconsider earlier codes and emergent themes in the light of subsequent interviews. Themes were considered both in relation to the data set and in relation to individual projects; they were also compared with the themes identified from the other (mothers’ or volunteers’) data set. To ensure the validity of the analysis, one researcher (JM) undertook thematic analysis of all the transcripts and MR analysed a subset of the transcripts; codes and emerging themes were discussed and agreed. Both researchers were aware of the need to approach the analysis reflexively, putting aside their existing knowledge of the topic so that the analysis remained close to participants’ accounts, and acknowledging the potential impact of their own perspectives as White, UK-born women with children.

## Results

This section of the paper describes the study participants and the themes that emerged from the interview data related to perceptions of peer support, reflecting the key issues across and between the different models of peer support in action.

## The participants

A total of 47 volunteer peer supporters and 42 women who had received peer support during pregnancy and early parenthood were interviewed.

The volunteers were a very diverse group of women. Twenty-nine were born in the UK and eighteen were born abroad in Africa, Europe, South Asia, North America, the Caribbean and Australasia. Twenty-three volunteers were White, fourteen were Black, nine were Asian, and one was mixed race. They had between one and eight children (median two) and ranged from young mothers who had never worked (the youngest were 22 years), to professional women in their forties and fifties with adult children. Sixteen volunteers spoke English as an additional language.

The supported women interviewed were similarly diverse in their backgrounds and experiences. Twenty-two were first time mothers (one of whom had twins), fifteen had two or three children, four had four or five children and one was a grandmother caring full time for her granddaughter. The youngest was 19 and the oldest was over 40 years of age. Twenty-one were single parents. Thirty- one were born overseas, coming from Africa, Eastern Europe, South America, South Asia, East Asia, the Caribbean and the Middle East; eleven were born in the UK. Eighteen were Black, thirteen were White, three were Asian and eight were from ‘other’ ethnic groups. Twelve had English as their first language. They had experienced a range of challenging circumstances including isolation, depression, anxiety, poor physical health, learning disabilities, HIV, domestic abuse, childhood sexual abuse, rape, people trafficking, motherhood at a young age, the asylum process, detention, insecure immigration status, homelessness, poverty, detention or imprisonment of a partner, having children taken into care, and the death of a child.

## Perceptions of peer support

The objective of the study was to explore a diverse group of mothers’ and volunteers’ perceptions of peer support in which they had been involved. In analysing both the mothers’ and volunteer supporters’ transcripts, three overarching themes were identified which related to the ways in which the support was experienced and understood. These were: ‘What is peer support?’, ‘Who is a peer supporter?’, and ‘The peer support relationship’. The themes are listed in Table 2. Participant quotations are used to illustrate these with ‘V’ and unique reference number indicating a volunteer supporter, and ‘M’ and unique reference number indicating a mother.

**Table 2:** Perceptions of peer support

Overarching themes	Themes
What is peer support?	Befriending or mentoring
	Responding to the individual
Who is a peer supporter?	‘Someone like me’
	Valuing difference
The peer support relationship	‘A friend’ or a ‘professional friend’
	Building a relationship of trust
	Avoiding dependency
	Managing endings
	Different from professionals

## What is peer support?

This overarching theme reflects participants’ understanding of the approaches that were implicit in the different models and the way that they as individuals provided or received peer support. The themes that were identified in relation to perceptions of models of peer support were: ‘befriending or mentoring’ and ‘responding to the individual’.

## Befriending or mentoring

This theme explores participants’ perceptions of the nature of peer support. At the heart of all the projects was the establishment of a confidential relationship of trust between volunteer and mother, but there was a broad spectrum of how this worked in practice, both between projects and between individual volunteers in the same project. At one end were projects where the volunteers understood their role as being largely about ‘befriending’ pregnant women and new mothers who needed social support and affirmation :‘*Sometimes it’s very general companionship, and if you like friendship - someone to actually discuss the pregnancy with…. company and a reassurance”* (V005). At the other end of the spectrum were volunteers in projects inspired by the ‘mentoring’ model which emphasised structured visits and setting goals [30]:

*We help (women) to understand how you can get to the goal that you want by taking smaller steps and listen to the things that they’ve got going on in their lives or challenges they’ve had, and how to help them overcome those challenges and…we’re setting goals with her and timeframes, “You need to do X, Y and Z in this period of time,” and then “How’s it going?…Have you managed to get X, Y and Z done?”* (V007)

This diversity was reflected in the terminology used to describe the volunteers, including ‘mentors’, ‘buddies’, ‘companions’, ‘community parents’, and ‘community supporters’. Table 3 shows some of the key differences between the ‘befriending’ and ‘mentoring’ models.

**Table 3:** Boundaries of peer support models

Befriending models	Mentoring models
Frequent visits (e.g. weekly)	Infrequent visits (e.g. monthly)
More spontaneous interaction during visits	More structured visits sometimes using facilitators or activities; setting and reviewing goals
Allowed to share contact details; volunteer can invite mother to own home	No sharing of personal contact details
Allowed to take own children on visits	Not allowed to involve own children
Allowed to offer direct emotional support, including reassurance, consolation and physical contact	Not allowed to offer direct emotional support
Flexible ending	Defined ending

Volunteers who understood their role as ‘mentoring’ were usually extremely conscious of the importance of boundaries to their role, which was not intended to include friendship*: ‘You’ve got to appear to be…very friendly so that the parent can actually open up to you, but you’ve got to have the boundaries…If one oversteps the boundaries then you lose the whole purpose of what you’re actually there for’* (V017). However, some volunteers found these boundaries emotionally challenging: *‘I can feel the tears coming and then I’ll have to get up and go out…You just want to get up and give them a hug. Or tell them it’s going to be alright…We’re not allowed to tell them what to do. Or give them a hug’* (V034).

Regardless of the volunteers’ understanding of their role as befrienders or mentors, for many of the supported mothers across all projects their primary purpose of seeking peer support was emotional connection: having an empathetic listener with whom they could share their feelings and problems: ‘*Because I feel upset and down and sometimes happy, sometimes sad, you know, I want to share my feelings somebody else’* (M013). This applied equally to mothers who had a husband or partner: ‘*It means I can talk to someone other than (my husband), 'cause I feel like I don’t want to offload him on all my problems’* (M026), and those who didn’t : ‘*It’s nice having someone to talk to because I don’t have that someone to talk to’* (M030).

None of the mothers in the ‘mentoring’ projects mentioned the goal-setting aspect of the support, and only one mentioned the structured activities. To some extent this discrepancy may have been because projects with a ‘mentoring’ approach sometimes used publicity materials that emphasised social support from another local parent: ‘*It was just the health visitor [told me] the basics of someone to befriend, someone to come, just chat with about anything, whatever’s on your mind*’ (M023).

Even in the ‘mentoring’ projects, there were individual volunteers who responded directly to the emotional needs of the mothers they supported. The plurality of approaches is illustrated through the accounts of the following two volunteers from the same project:

*The whole purpose is to empower people to be independent… you’re not a friend, you’re kind of an interim, you’re someone they’re not frightened of, and you’re signposting…. You establish what they might need… you find out the information and then leave them to try and do it*. (V027)

*If maybe you find she is crying, I give a cuddle and I say, “Don’t cry, everything will be okay. Don’t worry about anything, we are here about you, so whatever you want, just you ask, if you want to take my number, you can have my number and call if you want to talk to me.”* (V029)

## Responding to the individual

A strong theme from material arising from all the projects was the personalised and responsive nature of the support. The starting point was establishing the mother’s needs and working out how best to meet them within the parameters of the project: ‘*When I first arrive I’ll just explain what we do - that we’re trying to help people’s lives be a bit easier, and give them examples’* (V027), or responding to her needs as they developed: ‘*I have been known to go out at daft o'clock and sit with a mum 'cause the baby’s been crying… absolutely screaming the place down… her moods were sort of getting jittery’* (V023).

In many projects the mothers appreciated how open and flexible their volunteers were in what they offered, for example visiting at times that were convenient, and being available on the phone in between visits: ‘*She makes time for me. Like if I need to ask her something, I’ll just ring her up and I’ll be like, “Can you talk?” and make an appointment and she’ll come to see you soon’* (M020). Mothers also described how the volunteer took a lead from the mother about the support needed and did not impose her own agenda: ‘*I always thought, “Are they going to go, ‘Why are you depressed?’” Start picking on my personal life, but they actually didn’t. (The volunteer) just went straight on at looking at, “What can we do for you?”’* (M006).

The downside of this flexibility, and encouraging the mother to ask for the support she wanted, was that what was being offered was sometimes unclear. Thus some mothers said that they did not know what they could reasonably ask for: ‘*I’m still not particularly sure what the scheme is actually there for, but I know what I’ve been using it for and that has really helped’* (M016). *S*ome were particularly shy about asking for practical support until it was specifically offered: ‘*If somebody offered me something I can accept, but I cannot (ask) “Please come with me somewhere’* (M013). Some were conscious of not wanting to intrude on an unpaid volunteer’s time, so if a volunteer suggested a long gap between visits they did not feel able to ask for more frequent visits:

*[The volunteer] would just suggest, “Oh when shall we meet again, in about four weeks?” and so it didn’t make me feel like I could say, “Actually…” I’m aware she’s a volunteer, you don’t want to take up too much time. (It would be better if there was) more frequency, maybe more regular time slot…then you don’t need to worry about you asking too much.* (M003)

## Who is a peer supporter?

This overarching theme encompasses a fundamental issue about the nature of peer support in relation to individual characteristics and identity, and the way that it is understood and operationalised by those involved. The contrasting themes that were identified in relation to perceptions of who can be a peer supporter were ‘someone like me’ and ‘valuing difference’.

### ‘Someone like me’

This theme explores participants’ perceptions of the importance of shared characeristics or identity. In some projects, volunteers were specifically selected because they had additional experiences in common with the mothers they were supporting (for example being young, belonging to an minority ethnic group, being an asylum seeker, living with HIV). Both the volunteers and mothers in these situations felt that the shared experience gave the peer supporter a unique understanding, enabling her to make a non-judgemental and empathetic approach to a mother who had often experienced stigma from the wider community, and meant that she could offer practical strategies for dealing with issues that she herself had experienced.

*That person already knows that you’re not patronising, that you do have a sense of how they feel… they’re more likely to open up more with somebody that’s been through a similar sort of issue than somebody that hasn’t.* (V031)

*I can [be] free to talk about myself without anybody saying, ‘Oh,’ or anybody still giving me names…I understand [the volunteers] were somebody like me too, we are both in the same shoe, they never blame me.* (M033)

Many volunteers expressed the desire to help other mothers avoid the difficulties they had faced themselves, and some explicitly linked their motivation for volunteering to their sense of real equality and connection with the women they supported: ‘*So there’s that wanting to help other people so that they don’t go through the same sort of feeling and struggles that I had or that they don’t last as long’* (V013). Some volunteers described how if they had not experienced the same situation, they would identify common ground or refer to other difficult life experiences to demonstrate that they understood adversity:

*I says, “Because I might not be in exactly the same situation as you’re in but I have been in a lot of situations in my life…Lived it the hard way and I’ve managed to pull through.”… I think it gives you a cred, that you’ve done something personally, whereas you might be trained to deliver something but you don’t have the kind of the scars to say you’ve earned the experience you’re talking about.* (V042)

Many described the challenge of using their own life experiences judiciously: talking about themselves could be useful to establish trust and credibility, but must not detract from the focus on the supported woman and the uniqueness of her experiences: ‘*You have to constantly be aware in the situation not to over-complicate it by talking about your personal life. Because it is such a relaxed situation…you can easily get into the mode of just telling them everything’* (V021).

### Valuing difference

This theme describes the value some participants put on dissimilarity. Some volunteers were from socio-cultural backgrounds very unlike the mothers they were supporting: for example they had the resources, social networks, language skills and confidence that the mothers lacked. They described how they mobilised their own social networks to obtain donations of things that the mothers needed, gave mothers lifts by car to appointments, and helped them to deal with authorities by making telephone calls or writing letters on their behalf. One volunteer suggested that her emotional distance from the difficult issues facing the mothers meant she could sometimes be more effective as a supporter:

*Some of the volunteers…aren’t far removed from the people we help. And they have an amazing skill in empathy. Which I don’t have. I can be sympathetic but I can’t put myself in their shoes… I think that actually might be one of the reasons that I manage to not get too emotionally involved.* (V027)

In some cases mothers described how they actively preferred to have a volunteer who was very different from them – for example, a person from a different community to avoid the risk of judgement and gossip: ‘*Do you know when you get Pakistani people with another Pakistani person, you can’t actually open up to them…And they asked me if I wanted an Asian lady and I goes, “I’d prefer White”’* (M020); or someone would be an effective advocate within an unfamiliar system:

*I can’t explain myself 'cause my English is not good, so you always need someone who can explain for you. And who knows the law. I think it helped me, 'cause [the volunteer] went with me, even to the GP, 'cause they don’t give you a letter if you ask them sometimes. But she explained everything to them properly and yeah, they give it.* (M037)

## The peer support relationship

This overarching theme concerned the way the relationship was constructed and managed by the mothers and volunteers. In the course of the participants’ reflections on the relationship, themes that were identified were: ‘A ‘friend’ or a ‘professional friend”, ‘Building relationships of trust’, ‘Avoiding dependency’, ‘Managing endings’, and ‘How peer supporters are different from professionals’.

## A ‘friend’ or a ‘professional friend’

This theme reflected the range of views supported mothers and volunteers held by supported mothers and volunteers about the nature of the peer support relationship, which largely, but not entirely, reflected the differing emphases of the specific projects. Some volunteers and the majority of mothers described their relationship as being like a mother, sister or friend. This this was most likely where the project focused on emotional support, set no time limit on the frequency or duration of contacts, gave intensive practical support at home following birth, or permitted volunteers to share something of their own lives with mothers (for example talking about their own experiences, taking their children on visits, or inviting mothers to their own homes).

*[The volunteer] is like my mum. Seriously, she [has] been like a mum to me. She is my friend, I can talk to her [about] whatever I want, I can meet her whenever I want… She is really friendly, she is patient, she will listen to you and I like everything about her.* (M037)

*Most of them they become like my sisters. They know it, it’s like they are part of my family. When they are very close to me I find it easier to support them. Yeah I know I’m supposed to be professional, but to be like friend as well, it makes it more easier for us to talk, because then they feel, “We can tell her anything.”* (V046)

Volunteers whose projects had stricter boundaries in terms of where, when, how and what support was offered, used different language: ‘*in a professional relationship*’ (V044), ‘*a professional friend*’ (V031, V035). Some of these volunteers found it easy to maintain emotional distance: *‘It’s a supporting role…So I don’t feel any need to get too emotionally involved’* (V018), but others struggled with this constraint: *‘These women are so lovely, and because they are in desperate need for a friend…it’s so hard not to become their friend’* (V030).

Some of the supported mothers understood these limits clearly: ‘S*he’s like your friend, obviously she’s not a friend*’ (M003); *‘We are just a support team, we are not friends’* (M043); although many did not: ‘*They’re not there to do a job, they’re there to be your friend’* (M029). A few chafed against the one-way nature of formal peer support which carried the implication that they needed a ‘service’ or that there was no mutuality in the relationship. Some volunteers described how mothers repositioned the relationship by refusing formal meetings in favour of social encounters, or reciprocating hospitality:

*I think she’s somebody who doesn't like to feel needy, so if she knows … I’ve got childcare and done all this to see her, it suddenly puts her in a position of someone needing a service… And so it’s difficult to know how the relationship’s going to carry on being supportive without her feeling she’s obviously being supported.* (V013)

*When she had her baby, obviously I was helping her and took her some dinners and stuff and she was really vulnerable, but a couple of months later when she was more on her feet she came round and brought me a dinner which she made.* (V014)

Several of the mothers described how their volunteers’ active listening support was more helpful than the social support of ‘real’ friends and family, because it was non-judgemental and non-directive: ‘*She is a non-judgemental person, it’s different to a friend because a friend would say, “Oh, why don’t you do this?” or, “Why [do] you worry so much?”* (M015). An interesting light was shed on the line between peer support and friendship by the experience of one mother who was allocated a volunteer who was already her friend. She found this constrained the effectiveness of the peer support relationship because she did not want to be seen to be imposing on her friend/volunteer and this meant that she did not ask her to accompany her to the hospital for her caesarean section, even though she would have liked to:

*If when she is my [volunteer] she is already my friend, sometimes we can [feel] shy and you can’t take the help…I don’t want to ask her because she is my friend and I don’t want to feel “I do this to you”… I don’t want to hear somewhere else I done this to her… I don’t want the other neighbour talk about it.* (M011)

## Building the relationship of trust

This theme concerns the ways in which volunteers quickly won the trust and confidence of mothers, many of whom were in vulnerable situations. Some mothers described how they had been suspicious at first about the volunteers’ motivation, but had been persuaded to trust them by their persistence, reliability, lack of agenda and by their volunteer role:

*Once I knew that that person wasn’t there to invade my space…and it weren’t going to be, “I am coming at a set time on this week and doing this…” It was very much a more informal thing you know, like “When’s best for you?” And admittedly I’ve cancelled her quite a few times and most people would have said, “Do you know, sod you, you’re on your own.” But she hasn’t.* (M016)

*[The volunteer] was like on 24 h call outs, she would say, “Phone me.” She could come to my place six in the evening, and I thought, “This lady doesn't know me and she is just volunteering to do this. Why is she sacrificing her own personal time? …She wouldn’t be bothering unless she really cared.”* (M006)

For some mothers, especially those with a physical or mental health condition that their volunteer shared, the key ingredient in building trust was the volunteer’s willingness to be open about her own experiences : ‘*I was more free to [the volunteer] because she was quite open to me, unlike the midwife it was just a kind of hospital routine’* (M031). A few mothers expressed strong views about how they trusted the experiential knowledge of other mothers over the academic knowledge of health professionals without children:

*Some midwives they don’t have any kids. And when I ask about the breastfeeding they are trying to answer me as the profession or as they read in the book or learn in the college, but when I ask another mum already they had practical experience. That’s why they can answer you better than non-practical one… that is more acceptable and helpful for me.* (M036)

Practical support could be another important element in building trust with vulnerable mothers. Although improving mothers’ material situation could be an end in itself, or a recognition that very disadvantaged mothers were not able to engage fully with their pregnancy or parenting when they were overwhelmed with other crisis issues, volunteers also described how appropriate practical support could be a way of demonstrating understanding of the mother’s real concerns: ‘*If [a woman] said, “I’m going to be evicted tomorrow” … and you say, “Oh, have you got a bag packed for hospital?” - it just looks ridiculous…We can recognise the priority and we can recognise that it needs to be acknowledged’* (V006).

Some mothers also described how they had experienced practical support as a demonstration of genuine caring and how it had been a mechanism for creating trust:

*I developed…fever and one day my body just shut down…[the volunteer] literally ran my shower, she took off my clothes, she put me in the shower, she creamed my skin, dressed me, put me to bed, she made me some soup, she stayed with me for about four hours… …They are like a part of my family… because they they’ve treated me no different to somebody that as far as I’m concerned I would consider a friend.* (M007)

### Avoiding dependency

This theme reflects the challenges associated with avoiding dependency, when empowerment of mothers and building up their self-confidence was a specific aim of most projects. The volunteers were usually very aware of navigating the line between having a close and supportive relationship and creating dependency: ‘*My instinct would be to be a little bit more motherly, but the whole point is I want to empower her. I don’t want her to become dependent on me*’ (V016). However, several volunteers in projects where the boundaries were less strictly defined had experienced situations where a mother with high support needs had become dependent on them to an extent that they recognised as unhelpful. They described how they responded with greater clarity about the limits of the role and a stronger focus on enabling the mother to find ways of resolving her own problems:

*I gave a lot of time initially and would do everything to support her that I could… However, once she’d gone to the emergency accommodation and we had everything for her …the power went out in the emergency accommodation and the first person she contacted was me. … I hadn’t realised how dependent she was actually getting on me, so that made me draw the line and say, “I’m not able to support you with this.”…It was a hard conversation but she realised that there’s more than just me out there to help her…I thought I was being supportive but I didn’t realise I was not allowing her to solve her own problems.* (V004)

One project that worked with pregnant women with very complex needs avoided 1:1 support because of the risk of dependency on an individual volunteer; instead they mainly supported women in small teams and encouraged women to ‘bond’ with the organisation. In two projects that offered peer support groups as well as 1:1 support from a volunteer, the 1:1 support was envisioned as an initial intense phase that would build a mother’s confidence to attend the groups, at first with the volunteer, and then on her own:

*Initially it was one-to-one but now I am trying to encourage the women to come out to the drop-in sessions….I’ve said to her “I’ll meet you there, and if you do want to see me then you can either ring me or come and see me at this group,” so I’m trying to get her out and about… because I think I’ve done what I can at home.* (V022)

### Managing endings

This theme relates to the ways in which, when and how the support was brought to an end affected participants’ understanding of what the relationship meant. In some projects, mothers were unaware when the support would end, or believed that it would continue until they felt ready to end it: ‘*I think it’s as long as you want it. I’m not too sure*’ (M023). Many of the mothers described ongoing social contact as their ideal, validating the genuineness of the relationship: ‘*I would like we are friends forever, friends for life’* (M001); some volunteers also felt this: ‘W*hen you get to know them it’s like they are part of you…It’s like this thing should not have an ending’* (V048).

Other volunteers and mothers described how the relationship could come to a natural end or evolve into occasional social contact as the mother became more confident, resolved her problems and established her own social network:

*Most of the women that I’ve supported, once they’ve had their child the relationship becomes different… I shouldn't say you’ve served your purpose, but you have, in a way. Because they’re onto a next stage now…You go from seeing someone regularly to gradually not seeing them regularly. And then you basically see them now and again.* (V052)

*Already I started making friends in the women’s centre…[the volunteer] and I still talk, we greet …but for now, I mean the kids are grown.* (M028)

There could, however, be tension between ‘letting go’ as a goal and taking a more gradual approach to an individual’s changing needs. In some projects, the length of the relationship was pre-defined and its ending could leave the woman with a feeling of loss if she was not ready, as shown by this mother’s description of what it was like to finish with her volunteer: *‘Hard. 'Cause I’ve got to know [her] and… yeah, she’s sort of grown to know my family really, so she’s been like part of the family almost.’* (M030).

## Peer supporters are different from professionals

All of the mothers had experience of using the maternity services and many also had experience of contact with social workers or mental health professionals, and this theme is about the differences all of them (and all the volunteers) consistently described between professional and peer support. Many described themselves as being open or ‘free’ with a peer supporter in a way they could not be with a professional:

*(Because she’s a volunteer) it makes me more free…to talk to her….With social working and stuff, they tend to make you scared…of what they will do, they can take your kids. I know I’m not a bad mum but some things can go wrong at times. Yeah, so anything that I think I’m not doing right I will say to [the volunteer] and she will put me in the right way.* (M012)

Many mothers described how peer support differed because it was grounded in a relatively long term relationship, the volunteer knew them personally, and she offered flexible, personal support. They contrasted this with professionals, who were time-pressed, had a pre-existing agenda, had fixed hours and responsibilities, had no sustained relationship, and were mainly interested in the baby not the woman:

*They’ve actually got their heart in it. You know, they’re not doing it just for a job for money…they’re not like, “Oh, it’s just my job.” Clock in at Monday 9 o'clock, Friday at 5.30 I’m gone. But, you know, you can text [the volunteer] whenever and she doesn't care.* (M026)

*All the other professionals are so regulated, it’s nice to have one who is just there… for you rather than, you know, the babies.***(**M003)

The other important difference that women described was that peer support from a volunteer had an intrinsic emotional meaning: someone cared enough about you to give you her own time:

*I think with volunteers, they’re so much better than professionals because they’re doing it because they want to. I think it’s something inside yourself as well, you know that that person is there in their own time and there because they want to be, not because they have to be.* (M016)

All the volunteers had a similar perspective on what distinguished peer support from professional support, citing their understanding of the woman based on their relationship, trust, flexibility, available time and their empowering role which could give women a sense of agency over their own lives:

*I’m not coming in with all this advice, I’m not like this health professional who’s going to tell her to do this and do that with her child, I’m just there to support her in whatever decision she kind of wants to make. So I think she felt more like in control, whereas with other services, like health professionals, you don’t feel in control 'cause you feel like they’re telling you what to do.* (V035)

One mother described how she saw her peer supporter positioned ‘between the NHS and a friend’, providing something unique:

*If I want to get some professional suggestions I should contact GP or midwife, but they don’t have enough time to understand… your situation personally. If I want to get emotional support from friends, friends can give me suggestion but their suggestion may not fit for you. I think the volunteer provides a package of solutions, choice, and they told you what’s pros and cons, and you make decision which is right for you. There is no push, no demand… It’s kind of between the NHS and a friend.* (M043)

## Discussion

This research shows the plurality of models of volunteer pregnancy and early parenthood peer support currently operating in England, and the diversity of perceptions of the construct, with three overarching themes of ‘what is peer support?’, ‘who is a peer supporter?’, and ‘the peer support relationship’.

Our findings show the challenge of precisely defining volunteer peer support during pregnancy and the early months of parenting. Volunteers described working within a range of official models, from structured visiting with an emphasis on empowering mothers to make decisions, solve problems, set goals and achieve positive behavioural change, to a more informal focus on providing social and emotional support through befriending, always alongside information about pregnancy, parenting and local services. It was apparent that some of the volunteers within an individual project might position their support activities at very different places on this spectrum; others volunteered within what they found to be clear (if sometimes challenging) organisational boundaries. This diversity, combined with the fact that most projects aimed to provide a flexible, needs-led response to each woman, and that some women did not feel able to ask an unpaid volunteer for more support than was explicitly offered, meant that, consistent with Granville and Sugarman [3], the peer support projects sometimes struggled to convey exactly what they offered and some mothers were unsure what they could ask for or expect.

Dennis asserts that practical support is not a significant feature of peer support and that “peer support primarily occurs without the provision of instrumental support” [31]. However, this research has found that practical support was a very important feature of many pregnancy and early parenthood peer support projects, particularly those which supported very disadvantaged mothers. As well as being seen as a way of addressing crisis issues to enable the mother to engage with her pregnancy or parenting, volunteers and mothers also described it as a an expression of caring and a means of building trust.

There were different views about ‘who’ was a pregnant woman’s or new mother’s peer. Sometimes peer support was conceptualised as necessitating a shared experience of motherhood in the context of a specific illness or adverse social circumstances, with the unique understanding that shared experience could bring, in line with the understanding of ‘peers’ in other fields such as mental health [5, 32]. There were, however, many peer supporters who were peers as mothers but not ‘peers’ in this more specific sense, and yet whose support was highly valued, sometimes precisely because they had things that the mothers lacked - language skills, confidence, personal resources, social contacts, knowledge of English systems and independence from communities that were sometimes experienced as judgemental. This suggests that in the context of supporting transition to parenthood, as opposed to more specific support for people experiencing mental or physical illness, a shared experience of motherhood may for some be a sufficient basis for a ‘peer support’ experience, even in the absence of other shared characteristics.

These differences over ‘who’ was a pregnant woman’s or new mother’s peer were also manifest in the question of ‘what’ the relationship was and meant. For some volunteers and mothers it was a genuine bond of friendship that sometimes felt as strong as a family relationship; it was long term and open ended, and sometimes acquired a reciprocal element. For others it was a purposeful friendliness with clear boundaries that would be formally terminated at a pre-determined point if it did not naturally fade away. Although there was an overlap, volunteers were much more likely than supported mothers to understand the relationship as ‘professional friendship’, and mothers were much more likely to use the language of *‘friend’*, *‘sister’* or *‘mother’*. Peer support was, however, also seen as a unique relationship because unlike a ‘normal’ friend or relative, the peer supporter did not tell the supported woman what to do, but as in Small et al. [22], supported her through active listening. In contrast to the volunteer doulas surveyed and interviewed by Spiby et al. [16], who were required to cease contact 6–12 weeks after birth, many of the volunteers in these projects were able to exercise a choice about the extent of their emotional engagement in the relationship and whether it continued as a social friendship after support had ended.

Although volunteer peer support was perceived in a variety of ways by those giving and receiving it, there was a defining common element. Each model of peer support was ultimately a structure within which a confidential, meaningful and potentially sustained relationship of trust could be built between the volunteer and the supported mother. Dennis’ concept analysis [31] of peer support within healthcare cautions that the training of supporters, while necessary, risks turning them into ‘paraprofessionals’ with an allegiance to the healthcare system, but that “the amount of training required to create a paraprofessional is uncertain”. Although there was wide variation (between 8 and 75 h) in the initial training given to volunteers at these nine peer support projects, all volunteers and mothers in this study stated clearly that the peer support relationship was fundamentally different from that of a health or social care professional because the woman felt that she was known and respected as an individual and the support was flexible, personal, non-judgemental and focused on her wellbeing. Furthermore, the volunteer made a gift of her time, affirming the mother’s worth.

A key strength of this research was the inclusion of 89 participants with very diverse personal circumstances (including some extremely disadvantaged backgrounds), from nine volunteer peer support projects around England, and the parallel analysis of the experiences of volunteers and mothers, enabling reflection on both different models and the perspectives of those giving and receiving peer support. There were also some limitations. Firstly, the numbers of participants from individual projects ranged from three to thirteen, thus there are limited qualitative data from some projects. Secondly, participants were contacted through the project co-ordinators which was essential to gain the trust of some very vulnerable women, but meant that the researchers were not aware of how many declined to participate at that early stage. Thirdly, one mother’s interview was informally interpreted by her peer supporter at her request, so her comments about the support she had received had to be considered in this context (she is not quoted in this paper). Fourthly, although we requested that mothers be approached to take part after their peer support had ended, in practice some mothers were included whose peer support was ongoing, because of the challenge of arranging interviews with women whose accommodation and sometimes immigration status were precarious. This meant that some participants were not able to reflect on the whole lifecycle of a peer support relationship.

## Conclusion

A variety of models of volunteer peer support are offered to pregnant women and new mothers in England. These peer support projects differ in what support they offer, to whom, by whom, how frequently, for how long, and how they train their volunteers. Those giving and receiving volunteer peer support hold diverse views about the nature and purpose of their relationship. However, all forms of peer support that match a mother with a volunteer or volunteers create a structure for socially meaningful relationships of trust to occur. Volunteer peer supporters therefore have the potential to connect with and give support to vulnerable and marginalised mothers, and to enable them to access services, in ways that complement the work of health professionals. They have the potential to improve physical outcomes for mothers and babies by increasing the uptake of maternal and child health services, and may improve emotional outcomes by forming relationships that reduce feelings of isolation and stress and increase feelings of empowerment and capability. The impact of peer support on pregnant women and new mothers will be explored in subsequent papers.

The range of models, and the diversity of perceptions and practices between and within peer support projects, means that there is no agreed or common understanding of volunteer peer support during pregnancy and early parenthood. It is important that individual projects give clear information about what kind of model is in operation and what they actually offer to local health and social care professionals who may refer mothers, and to mothers who may benefit from support, without losing the flexibility and individuality of their response. It is also important for evaluation that the characteristics of the models are fully described to enable meaningful comparison.
